# Case of Relapsed, Metastatic Cutaneous Squamous Cell Carcinoma With HER2 Mutation Treated With Trastuzumab

**DOI:** 10.1002/ccr3.70726

**Published:** 2025-08-29

**Authors:** Yae Kye, Joseph Marshalek, Dalton Wise, Shi Peng, Andrew Hwang

**Affiliations:** ^1^ Department of Internal Medicine Harbor‐UCLA Torrance California USA; ^2^ Department of Internal Medicine, Division of Hematology and Medical Oncology Harbor‐UCLA Torrance California USA; ^3^ Department of Pathology Harbor‐UCLA Torrance California USA

**Keywords:** cutaneous squamous cell carcinoma, ERBB2, HER2, metastatic squamous cell carcinoma, receptor tyrosine kinases, trastuzumab

## Abstract

Cutaneous squamous cell carcinoma (SCC) is a common skin cancer with a favorable prognosis when diagnosed at an early stage and fully resected. While the risk of recurrence and metastasis is relatively low for most patients, management of unresectable or metastatic SCC can be challenging due to poor responses to chemotherapy and therapy‐related toxicities. The HER family of receptor tyrosine kinases has been associated with cutaneous malignancies including SCC. However, the pathogenic influence and targetability of HER2 in cutaneous SCC are not well defined. Here, we describe a case of a patient initially presenting with metastatic SCC who underwent primary tumor resection, radiation, and four systemic therapies due to disease progression before her disease was found to harbor an ERBB2 (HER2) mutation; she was subsequently started on HER2 targeted therapy with trastuzumab and achieved a significant sustained response to treatment. This case demonstrates the clinical challenges of treating advanced SCC and highlights HER2 as a potentially targetable driver mutation. Future research is needed to evaluate the association of the HER family and cutaneous SCC, as HER2 may represent an effective therapeutic avenue for patients harboring this mutation.

## Graphical abstract

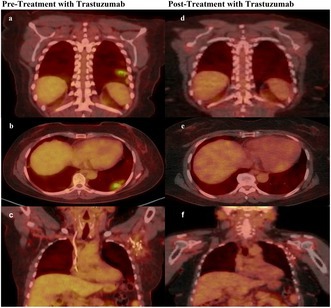

**Abbreviations**
cSCC: cutaneous squamous cell carcinoma
ICI: immune checkpoint inhibitor
LN: lymph node
RTK: receptor tyrosine kinase

**Summary**

- Managing metastatic cutaneous squamous cell carcinoma (cSCC) is clinically challenging due to poor response rates and therapy‐related toxicities.
- The HER family of receptor tyrosine kinases is associated with cSCCs; however, its pathogenicity and targetability are not known.
- This case successfully demonstrates HER2 as a potential therapeutic target for recalcitrant cSCCs.

## 1 Introduction

Cutaneous squamous cell carcinomas (cSCC) are one of the most common cancers, accounting for 20% of cutaneous malignancies [1, 2]. Most cSCCs are diagnosed at an early stage and treated surgically with good outcomes [2]. Approximately 3%–5% of cSCCs develop nodal metastases, which are associated with higher rates of recurrence despite resection and worse prognosis [1, 3]. For unresectable and/or metastatic disease, non‐surgical treatment modalities include radiation, immunotherapy, chemotherapy, photodynamic therapy, and topical treatments. The combination of these non‐surgical treatment modalities, such as cemiplimab and localized radiotherapy, has shown to decrease tumor burden and disease progression [4]. However, despite these options, advanced cSCC remains challenging to treat, as a significant proportion of these patients will respond poorly and/or have disease relapse [2, 5, 6].

The epidermal growth factor receptors HER2, HER3, and HER4 are receptor tyrosine kinases (RTK) that have been implicated in multiple malignancies [7, 8]. This particular family of RTKs is known to regulate keratinocyte proliferation and differentiation, highlighting its oncogenic potential [9]. Prior studies have associated both HER2 and HER3 overexpression with cSCC as well as other cutaneous malignancies [7, 8, 10, 11]. Despite these findings, the therapeutic potential of the HER family in cSCCs has yet to be fully characterized. Here, we report the case of a patient with stage IV cSCC with lymph node and lung metastases who was refractory to 4 prior lines of therapy, subsequently found to have an ERBB2 (HER2) mutation, and then was switched to trastuzumab with continued response following a year of treatment.

## 2 Case History

**FIGURE 1:**
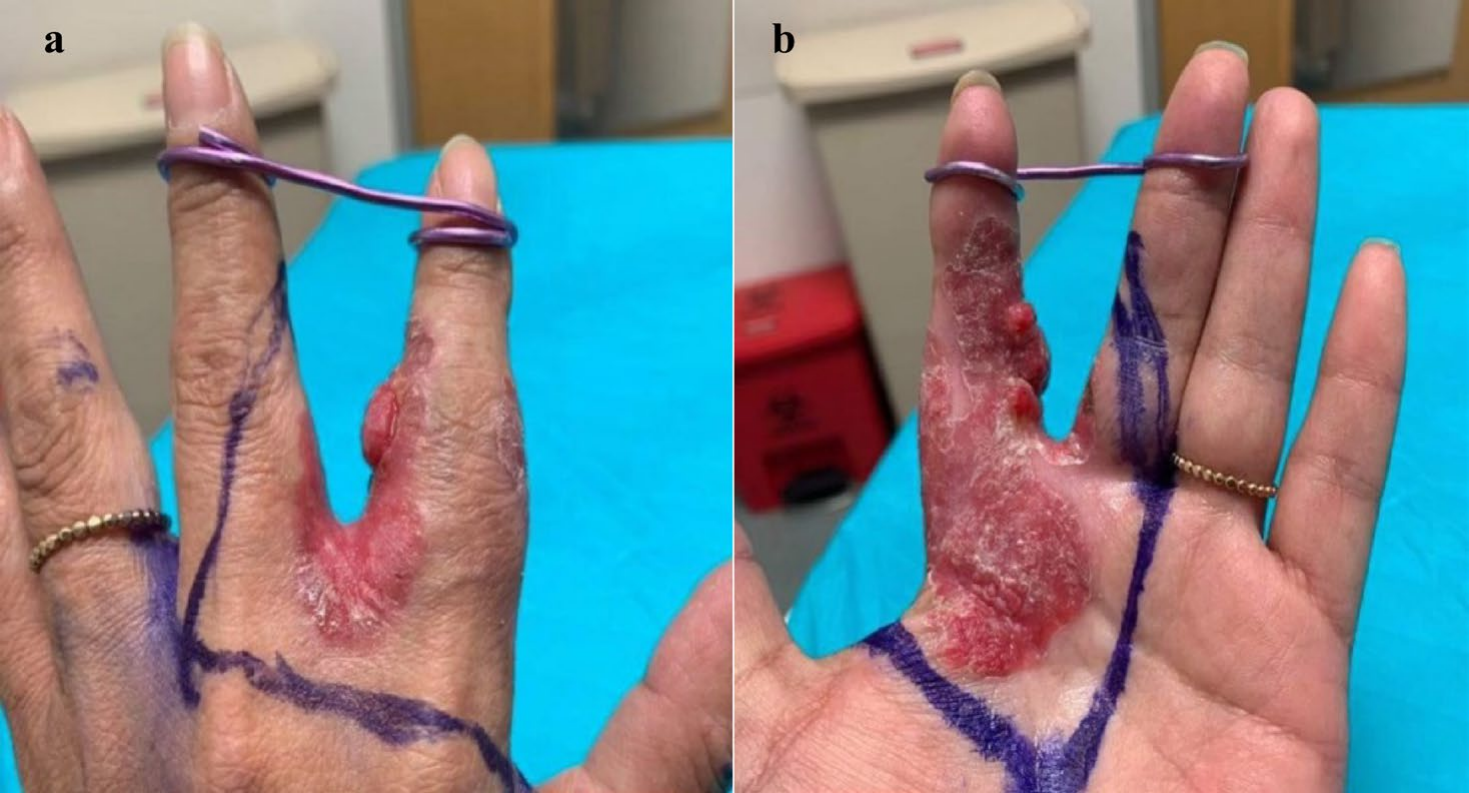
Clinical photographs of cutaneous SCC involving the L hand. (a) One‐cm pink‐red nodule within a pink‐red plaque with well‐defined borders involving the second‐third interdigit space on the L dorsal hand. (b) Multiple pink‐red papules within a pink‐red, eczematous well‐circumscribed plaque involving the second digit extending to the left palm. SCC, squamous cell carcinoma.

A 56‐year‐old female presented to our Oncology clinic with a 1‐year history of poorly differentiated metastatic cSCC that was previously treated with carboplatin + paclitaxel and pembrolizumab (PDL1 expression 20%). On examination, the patient had a nodular plaque involving her left second finger that was present for over 20 years (Figure 1).

## 3 Investigations and Treatment

**FIGURE 2:**
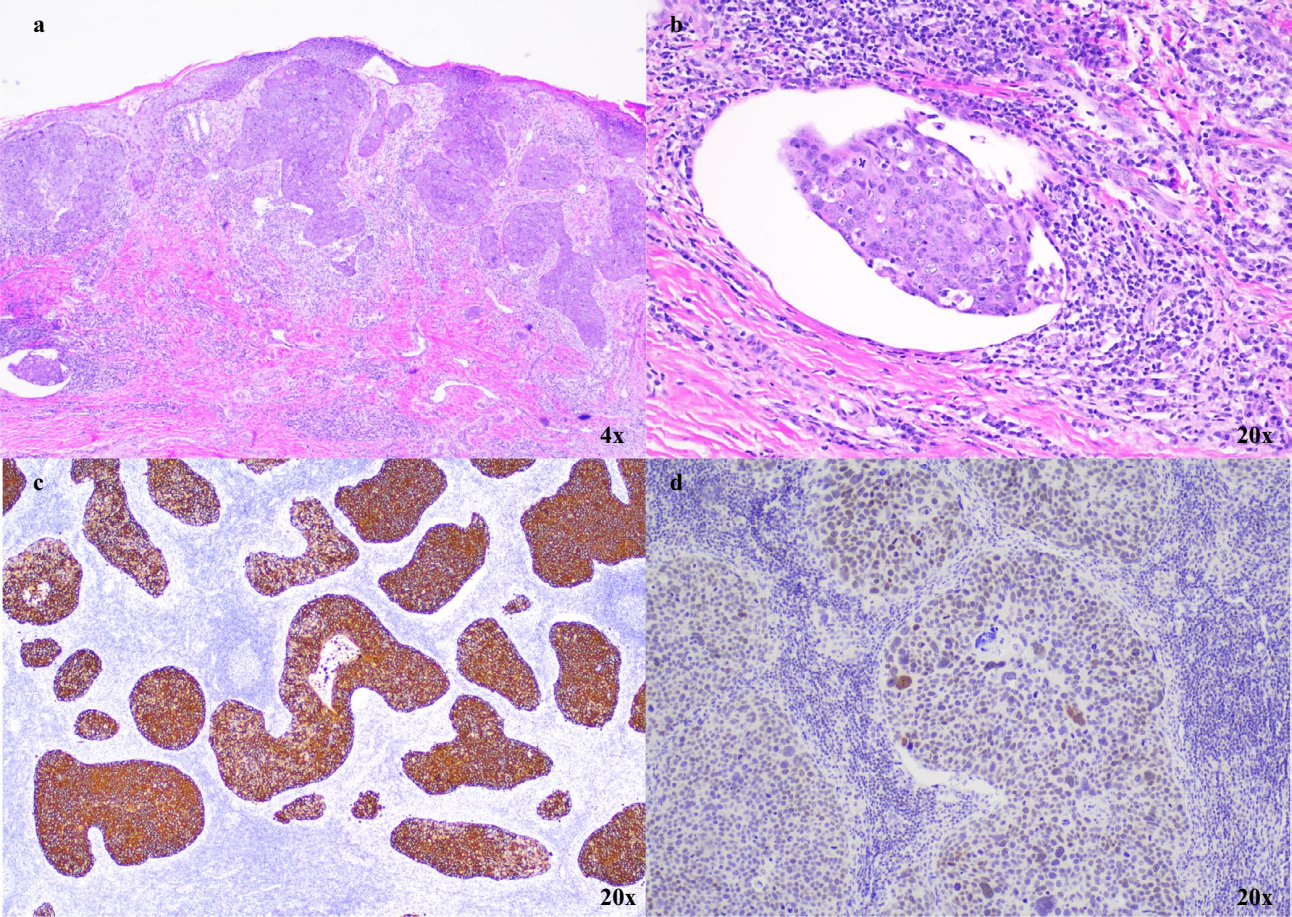
Histopathology of primary (a, b) and metastatic lesions (c, d). (a) H&E 4X of cutaneous primary lesions: Low power of the proliferation of invasive atypical keratinocytes arranged in nests with connection to the epidermis. (b) H&E 20X of cutaneous primary lesions: High power image of lymphovascular invasion with a small nest of SCC within a dilated vessel. (c) IHC stain of Cytokeratin 5/6 in Lymph Node 4X: Strongly staining Cytokeratin 5/6 highlighting metastatic SCC in a lymph node. (d) IHC stain of GATA 3 in Lymph Node 20X: Patchy positivity confirming skin as the primary location of the tumor as lung SCC would be negative for GATA‐3. IHC, immunohistochemistry; SCC, squamous cell carcinoma.

**FIGURE 3:**
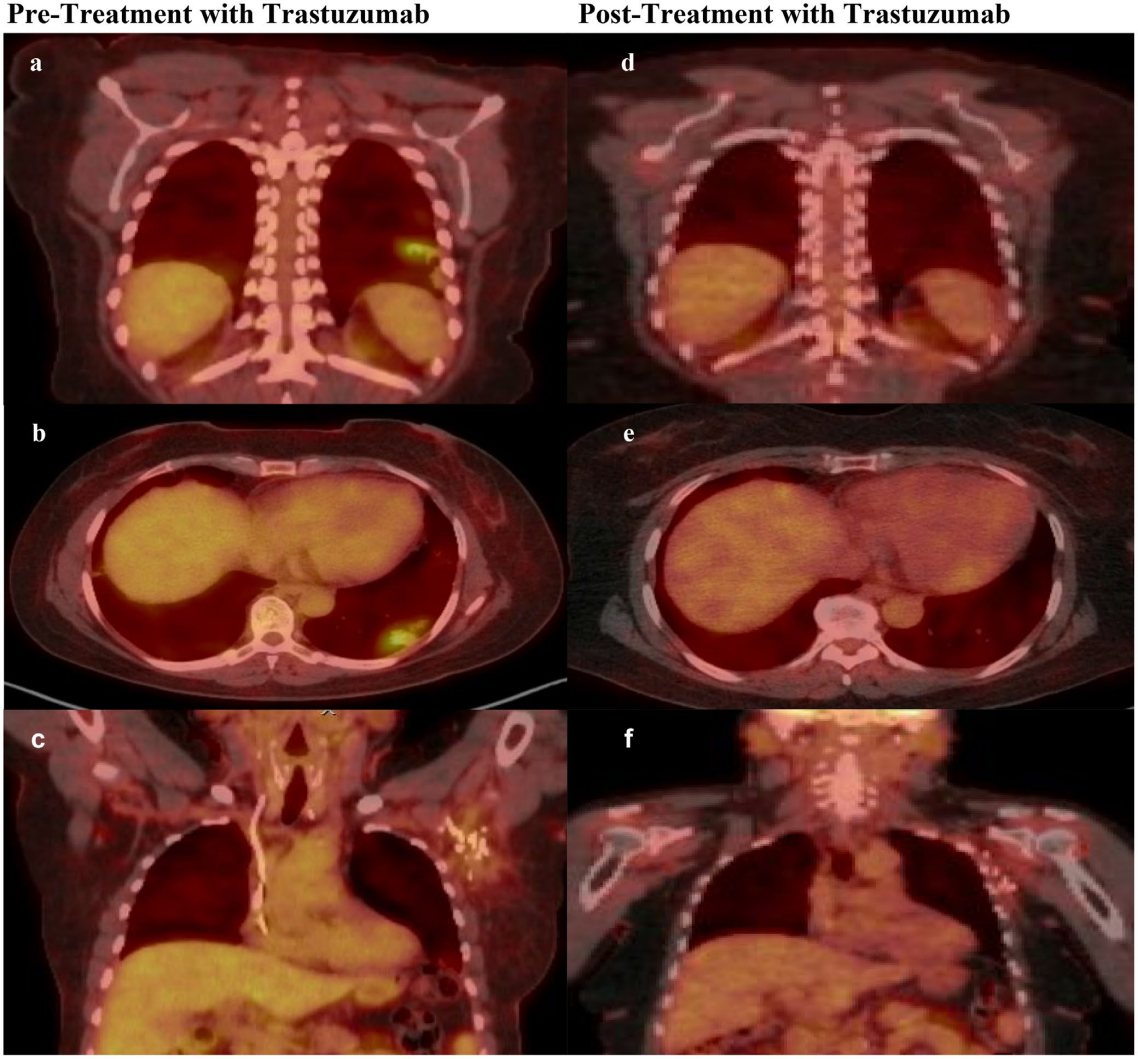
PET‐CT of lung and lymph node metastasis pre‐ and post‐trastuzumab treatment. (a, b) FDG avidity showing left lung (4.1 cm, SUV max 5). (c) Left axillary lymph nodes pre‐trastuzumab treatment. (d, e) Absence of FDG avidity in left lung. (f) Left axillary lymph nodes post‐trastuzumab treatment.

Skin biopsy was taken and confirmed the presence of cSCC. PET‐CT and MRI imaging showed a metabolically active plaque‐like soft tissue mass of the left index finger as well as multiple enlarged left axillary lymph nodes (LN) and nodular foci within the lungs consistent with metastatic disease. The patient underwent surgical resection including left index finger amputation, left third finger soft tissue resection, and left axillary LN dissection. Histopathology results showed invasive cSCC with lymphovascular invasion of the left index finger and metastatic cSCC in 13/31 LN (Figure 2a,b). Further immunohistochemistry of the axillary LN identified the cSCC as the primary tumor origin (Figure 2c,d). The patient subsequently received 19 cycles of cemiplimab and radiation to the left axilla (total 6600 cGy in 33 fractions), with eventual disease progression presenting as a new right lower lobe lung metastasis (Table S1). She then received five cycles of capecitabine with improvement of her right pulmonary lobe opacities but then subsequently developed new FDG‐avid (SUV max 5) confluent nodular opacities in the peripheral left lower pulmonary lobe measuring 4.1 cm (Figure 3a,b) and in the left axilla (Figure 3c). Next‐generation sequencing (Tempus, Chicago, IL) revealed an ERBB2 (HER2) mutation, and she was then started on trastuzumab (Table 1).

**TABLE 1:** List of mutations identified in next‐generation sequencing with potential treatments.

Mutation	Description	Potential treatments
ERBB2 (HER2)	Copy number gain	Trastuzumab Neratinib (Pan‐HER TKI) Lapatinib (Pan‐HER TKI)
ZNF750	p.R134fs Frameshift—LOF	
TP53	p.R337L Missense variant—LOF	
TERT	c.‐146C>T Variant—Promoter mutation	

*Note:* Adopted from TEMPUS.

Abbreviations: LOF, loss of function; TKI, tyrosine kinase inhibitor.

## 4 Outcome and Follow‐Up

A 3‐month follow‐up PET‐CT showed a good response to treatment, with size reduction and reduced FDG avidity in her lung and nodal metastases. After 12 months of treatment, she had radiographic resolution of her previously FDG‐avid 4.1 cm left lower lobe lung lesion (Figure 3d,e) and left axillary lymph nodes (Figure 3f) with stable low FDG uptake in the mediastinum (SUV max 2.28). Overall, the patient had a near‐complete response to trastuzumab and presently remains on treatment without any significant cardiac or other adverse effects.

## 5 Discussion

Historically, metastatic cSCC has been associated with poor prognosis and limited effective therapeutic options. In studies of advanced or metastatic cSCC prior to immune checkpoint inhibitors, response rates of around 30% were observed with chemotherapy, with median overall survival less than 1 year [1, 12]. Immune checkpoint inhibitors including cemiplimab and pembrolizumab are now first‐line treatment options for unresectable or metastatic cSCC. After progression on immunotherapy, cytotoxic chemotherapy (platinum‐based, paclitaxel, and fluorouracil) or cetuximab can be used, but response rates are low and not durable. Expanded therapeutic options are therefore needed. There is a paucity of research and guideline recommendations regarding molecularly targeted therapies for cSCC.

In this case of relapsed metastatic cSCC, the patient received two checkpoint inhibitors (pembrolizumab and cemiplimab), two chemotherapy regimens (carboplatin + paclitaxel and capecitabine), radiation, and multiple surgeries; this illustrates the highly refractory course that can be seen with advanced cSCC. Next‐generation sequencing revealed a copy number gain in the ERBB2 (HER2) gene. Trastuzumab was initiated in the fifth line setting, and the patient achieved systemic disease control with a near complete response, which is ongoing at 12 months.

The overexpression of the RTKs in the HER family, specifically HER2 and HER3, has been identified in cSCC [7]. Prior studies of cSCC human tissue samples and in vitro cSCC cell lines have found HER2 and HER3 overexpression, suggestive of a potential role in tumorigenesis [7]. From our review of currently published literature, this case would be the first report of trastuzumab use in metastatic cSCC. Given the plethora of HER2‐targeting therapies and tumor‐agnostic approval for trastuzumab deruxtecan, next‐generation sequencing and HER2 immunohistochemistry should be considered in cases of metastatic cSCC to identify potentially actionable mutations. Further research is needed to characterize the prevalence of HER2 mutations and overexpression/amplification in cSCC in order to determine the feasibility of trial design in this space. Of note, there were no patients with cSCC included in the DESTINY‐PanTumor02 trial, which demonstrated the efficacy of trastuzumab deruxtecan in HER2 overexpressing solid tumors [13].

Interestingly, our patient developed a cSCC in situ while on trastuzumab therapy, suggesting that the risk of local recurrence may be independent of systemic disease control and emphasizing the importance of close dermatologic monitoring. A prior meta‐analysis found that increased tumor depth and presence of perineural invasion were associated with a higher risk of local recurrence [14]. However, we were not able to assess these risk factors in our patient. Our patient remains on trastuzumab therapy and follows with Dermatology for routine skin examinations. Unfortunately, there are no standardized guidelines for follow‐up, and it is often individualized based on the patient's risk factors. Future studies may be designed to investigate the associated risk factors for disease recurrence and define guidelines for surveillance and survivorship care planning.

This case highlights the limitations in systemic therapeutic options and guidelines in managing advanced or metastatic cSCC as well as the utility of next‐generation sequencing as a diagnostic tool to guide and tailor treatment. It also highlights the therapeutic efficacy of trastuzumab in a recalcitrant metastatic cSCC with a HER2 mutation. Further research regarding the role of HER2 in cSCC is vital, as HER2‐targeted treatments may represent a promising therapeutic avenue in this otherwise poorly responsive and relapsing malignancy.

## Author Contributions

**Yae Kye:** conceptualization, data curation, formal analysis, investigation, methodology, resources, writing – original draft, writing – review and editing. **Joseph Marshalek:** conceptualization, data curation, supervision, writing – original draft, writing – review and editing. **Dalton Wise:** data curation, visualization, writing – review and editing. **Shi Peng:** supervision, validation, writing – review and editing. **Andrew Hwang:** conceptualization, supervision, writing – review and editing.

## Consent

Written informed consent was obtained from the involved patient. All information has been reviewed and approved to be published in this case report.

## Conflicts of Interest

The authors declare no conflicts of interest.

## Supporting information


**Table S1.** Overview of patient treatments and clinical response.

## Data Availability

The data that supports the findings of this study are available in the Supporting Information of this article.
