# Supplementary material for: Case of Relapsed, Metastatic Cutaneous Squamous Cell Carcinoma With HER2 Mutation Treated With Trastuzumab
Source: Clin Case Rep. 2025 Aug 29;13(9):e70726. doi: 10.1002/ccr3.70726 (PMC12396932; doi:10.1002/ccr3.70726)
Supplement: Supplementary file 1 — Table S1. Overview of patient treatments and clinical response. [file CCR3-13-e70726-s001.docx]

| **Supplemental Table 1.** Overview of patient treatments and clinical response | | | |
| --- | --- | --- | --- |
|  | **Treatment Course** | **Treatment Details** | **Response** |
| 1rst line | Carboplatin + Paclitaxel | - 6 cycles | Failed* |
| 2nd line | Pembrolizumab | - 6 cycles | Failed* |
| 3rd line | Surgical resection + Cemiplimab + Localized Radiation | - 19 cycles of cemiplimab  - IMRT 6MV to Left Axilla, for 53 days: Daily dose- 200 cGy, 33 fractions, Total dose- 6600 cGy | *Primary*: no recurrence *Metastases*: Progression, new right lower lobe pulmonary opacity |
| 4th line | Capecitabine | - 5 cycles | *Primary*: no recurrence *Metastases*: Progression, 4.1cm FDG avid nodular opacities in peripheral left lower pulmonary lobe |
| 5th line | Trastuzumab | - 16 cycles, with ongoing treatment | *Primary*: recurrence of SCC in-situ on left hand post 8 cycles *Metastases*: Stable, no evidence of metastatic progression or new metastatic disease |
| *Primary and metastatic response unknown as patient was treated at an outside hospital *Abbreviations*:  IMRT, intensity-odulated radiation therapy; 6MV, 6-megavolt photon beams; SCC, squamous cell carcinoma | | | |
